# Dietary flounder skin improves growth performance, body composition, and stress recovery in the juvenile black rockfish (*Sebastes schlegeli*)

**DOI:** 10.1186/2193-1801-3-235

**Published:** 2014-05-08

**Authors:** Jae-Ho Hwang, Sung-Ju Rha, Jae-Kwon Cho, Seon-Jae Kim

**Affiliations:** College of Fisheries and Ocean Science, Chonnam National University, Yosu, 550-749 Korea; Southwest Sea Fisheries Research Institute, National Fisheries Research and Development Institute (NFRDI), Yosu, 556-823 Korea; Department of Marine Bio Food Science, College of Fisheries and Ocean Science, Chonnam National University, Yosu, 550-749 Korea

**Keywords:** Fish skin, Protein source substitution, Growth performance, Body composition, Lysozyme activity, Stress recovery, Black rockfish

## Abstract

This study investigated the relationship between flounder skin meal (FSM) and vitamin C in mediating collagen biosynthesis. Based on the vitamin C requirements (150 mg/kg) of the black rockfish (mean body weight 10.05 ± 0.44 g), a vitamin C level of 400 mg/kg was selected, and 0, 5, 10, or 20% of the casein (purified proteins) in the diet was replaced with FSM. The feeding study was conducted for 8 weeks by using 4 experimental groups. The FSM supplementation resulted in improvement in growth performance, decrease of body lipids. Furthermore, it elevated the HDL-cholesterol levels and total protein content, reduced blood lipids, and led to rapid recovery in stress, which confirm the functionality of FSM with high collagen content.

## Introduction

Numerous studies have been performed investigated vegetable and animal proteins that could replace fishmeal in fish feed. In particular, there have been many studies on the use of vegetable protein sources such as soybean meal (Lee and Jeon 1996; Murai et al. 1982b; Robert et al. 1993), cottonseed meal, and rapeseed meal (Lee and Yoo 1996; Pham et al. 2005), which have a relatively stable supply compared to fishmeal, to replace fishmeal as a source of protein. However, plant resources are constantly in competition with livestock and human consumption, and the recent development of plant extract fuels such as bioethanol will eventually lead to an increase in the price of the plant resources usable as protein resources (Rena and Hasan 2009). The by-products of the processing of terrestrial livestock such as cows, chickens, and pigs could be used as animal protein sources, since they have a relatively high protein content and qualitatively similar amino acid composition to fishmeal, and are inexpensive and stably supplied. Various studies have been conducted on their use as protein sources to replace fishmeal in fish feed (Ai et al. 2006; Bai et al. 1998; Kikuchi et al. 1994; Kim and Bai 1997,1999; Lee and Lee 1998; Sato and Kikuchi 1997). However, the rise of safety issues due to serious infectious diseases like mad cow disease, swine fever, and avian influenza has gradually restricted the use of livestock by-products lately. Thus, as there are economic and safety issues with using terrestrial protein sources to replace fishmeal, securing economic and safe protein sources from marine products rather than terrestrial products is necessary. Many researchers have investigated by-products obtained from processing marine animal as potential protein sources, including shrimp by-products (Cruz-Suárez et al. 1993), tuna muscle by-products (Uyan et al. 2006), shrimp and fish by-products (Li et al. 2004), squid liver meal mixing soybean meal with by-products of squid processing (Kim and Bai 1997), fish bone and crab by-products (Goytortúa-Bores et al. 2006; Lee et al. 2010; Toppe et al. 2006), and fish by-products (Foster et al. 2005). Of the fishery by-products, even though fish skins obtained from the consumption of raw fish are a good protein source high in collagen content, by-products such as bones and internal organs are only partially used and mostly discarded.

Collagen, a main component of the extracellular matrix, is distributed in multicellular animals and is a protein that builds skeletons for the body structure. It also has many functions such as morphological changes and biological defenses in the proliferation, differentiation, and development of the cells involved in the maintenance of body or organ structures (Eyre and Wu 1987; Fessler and Fessler 1987; Kuhn 1987). In addition to type I collagen found in the dermis, bones, fins, scales, and muscles; type II found in notochords; type XI found in cartilage; and type V found in muscles, scales, and skin, a number of molecular varieties have been found in fish such as truncus (C-B) collagen present in the muscles and intestinal canals of cyclostomes, a special type of fish (Kimura 1997; Yoshinaka 1989). Until recently, collagen that originated from terrestrial animals and gelatin, the denatured products thereof, were considered to be safe and useful substances in various industry fields such as food, clothing, cosmetics, and cell culture. However, due to safety concerns following frequent outbreaks of mad cow disease, swine fever, and avian influenza, “marine collagen” derived from marine animals has gained attentions as a substitute (Lupi 2002; Schrieber and Seybold 1993). Recently, many researchers have investigated the physico-chemical properties of collagen and gelatin extracted from fish skin by-products of *Astroconger myriaster*, *Navodon modestus*, *Loligo bleekeri*, *Todarodes pacificus*, *Limanda aspera*, *Gadus macrocephalus tilesius*, *Thunnus albacares*, *Physiculus bacchus*, *Theragra chalcogramma*, and *Isurus oxyrinchus* to evaluate their industrial feasibility (Kim 1996; Kim and Cho 1996; Kim and Kwak 1991; Kim et al. [Bibr CR41][Bibr CR42][Bibr CR43][Bibr CR44]; Kwon et al. 2008; Park et al. 2009; Yoo et al. 2008).

In marine animals, the provision of feed mixed with hydroxyproline, a main amino acid of collagen, with vegetable proteins improved the growth of *Salmo salar* L. and increased the hydroxyproline content in the vertebral column (Aksnes et al. 2008). Consequently, positive *in vivo* functional effects of collagen depend upon the ingestion of its hydrolysates, peptides, or hydroxyproline and using fish skin containing collagen in abundance can provide these functional effects. Collagenase activity, especially in teleosts, was observed to be high in pancreatic tissues (Yoshinaka et al. 1978), indicating that the direct digestion of collagen is possible and collagen intake in fish is expected to have various physiological effects like those described above for terrestrial vertebrates.

Vitamin C, an essential cofactor in the production of hydroxyproline and hydroxylysine, which are important for collagen generation (Sandel and Daniel 1988), is known to be an essential nutrient with various functions in fish (Bai et al. 1996). Vitamin C also governs the formation of pyridinoline, which is known to cross-link materials during collagen maturation (Chan et al. 1990; Kim 2004; Nurad et al. 1981). In particular, the addition of vitamin C is reported to increase intracellular collagen synthesis when culturing 3 T6 fibroblast cells in mice (Kim 2006), indicating that vitamin C is significantly related to collagen. In fish skin, especially where there is a high content of the amino acids important for collagen composition like proline and glycine, adding vitamin C is anticipated to provide interactive outcomes with the collagen components in the fish skin.

Therefore, this study evaluated the usefulness of fish skin by-products as protein sources in mixed feed for fish farming. Based upon the results of previous studies, among *Paralichthys olivaceus*, *Sebastes schlegeli*, *Lateolabrax maculatus*, and *Pagrus major* (Cho et al. 2014), the skin of *Paralichthys olivaceus* was the thickest and was high in collagen fiber content and ASC. Therefore, it was powdered after hot air drying without special processes and directly provided to the black rockfish, and its potential as a protein source was investigated by elucidating the physiological characteristics of the fish. We also confirmed the synergistic effects of the collagen ingestion from the fish skin and vitamin C addition.

## Methods

### Experimental diet preparation

The skin of *Paralichthys olivaceus*, which has the highest farming yield and raw fish consumption in Korea, is easy to secure in large quantities due to its low use, thickness, and high content of collagen fibers and ASC, and was obtained from nearby fish markets. The fish skin was washed with fresh water and was subjected to hot air drying (50–60°C) followed by grinding via a high speed grinder (ZM-1000, Retsch Co., Japan) to prepare flounder skin meal (FSM).

Ingredients and proximate compositions of the experimental diets in response to FSM supplementation and the results of vitamin C analyses are shown in Table 1. Proximate analyses were carried out to evaluate the nutritional composition of the prepared diets, and the vitamin C content of the diets were analyzed using the 2,4-dinitrophenyl hydrazine (DNP) colorimetric method. Briefly, 5% metaphosphoric acid solution was added to a certain amount of the sample and ground by a homogenizer. The sample was then centrifuged and 5% metaphosphoric acid solution was added to the supernatant to make a final volume of 100 mL. One to 2 drops of 0.2% indophenol solution were added to 2 mL of the prepared solution and then 2 mL of thiourea-metaphosphoric acid solution and 1 mL of DNP solution were added sequentially followed by reaction at 50°C for 30 min. The sample was cooled down and 5 mL of 85% sulfuric acid solution was added. Absorbance was then measured at 540 nm and vitamin C was quantified by a standard calibration curve.

**Table 1:** **Ingredients and proximate composition of experimental diets with various levels of FSM**

Ingredient (%, DM)	FMS level (%)			
	Control (0)	5	10	20
White fish meal	41	41	41	41
Casein	20	15	10	0
Flounder skin meal (FSM)	0	5	10	20
L-ascorbic acid	0.04	0.04	0.04	0.04
Wheat flour	22.56	23.26	23.96	25.26
Squid liver oil	8.4	7.7	7	5.7
α-potato starch	3	3	3	2
Vitamin premix^a^ (vitamin C free)	2	2	2	2
Mineral Premix^b^	2	2	2	1
Choline Chloride	1	1	1	1
Total	100	100	100	100
Vitamin C in diets	391.35	411.37	416.77	409.56
^*^ *Proximate analysis*				
Protein	45.71	45.91	47.51	46.51
Lipid	10.11	9.21	10.91	9.91
Ash	8.01	8.21	8.31	8.31

Data are mean ± standard deviation of three group of fish (n = 3). Values with different superscripts are significantly different (*P* < 0.05).

DM dry matter.

ns not significant.

^a^ Vitamin premix (mg/g mixture) : retinol acetate, 0.81 mg; cholecalciferol_,_ 0.012 mg; vitamin E, 22.5 mg; vitamin K_3_, 2.5 mg, thiamine, 5.5 mg; riboflavin, 10 mg; pyridoxine, 6 mg; niacin, 37.5 mg; folic acid, 2 mg; biotin 0.05 mg; inositol 50 mg. All ingredients were diluted with alpha-cellulose to 1 g.

^b^ Mineral premix (mg/g mixture) : Mn, 3.2 mg; Zn, 3.2 mg; Fe, 3.0 mg; Cu, 0.36 mg; MgSO_4_, 100 mg; KCl (47%), 60 mg; Al(OH)_3_, 1.06 mg; Ca(IO_3_)_2_, 0.475 mg; CoSO_4_, 0.475 mg. All ingredients were diluted with alpha-cellulose to 1 g.

^*^Dry matter.

FSM is a high protein meal containing more than 80% crude proteins but is lacking in essential amino acids compared to fishmeal. When fishmeal is replaced by FSM, unknown factors present in essential amino acids and fishmeal may affect the experimental fish, making it difficult to evaluate the influences of FSM supplementation on experimental fish. Therefore, to maintain proper balances of the essential amino acids and to minimize the effects of the unknown factors in the fishmeal, white fishmeal (FF Skagen LT Supreme, Denmark) was fixed at the same level throughout the experimental diets. Casein, a purified protein, was used to control the protein content of each experimental diet. Squid liver oil (Ihwa, Korea) rich in DHA and EPA, which are essential fatty acids for the black rockfish, was used as a lipid source. Flours (CJ, Korea) and α-starch were employed as carbohydrate sources to control energy and bind the diets. To find the relationship between the level of vitamin C (Sandel and Daniel 1988), and FSM supplementation on the fish body, vitamin C (400 mg/kg) was added based upon the vitamin C requirements of the black rockfish reported by Bai et al. (1996). There were four experimental groups, including a control group with fishmeal and casein only and three experimental groups with 5, 10, or 20% of the casein replaced by FSM. The experimental diet was prepared 5 mm in diameter with a moist pellet maker (Sun Brand Industrial, Korea) and stored at −45°C until further utilization.

### Feeding trial, growth performance, and proximate analysis

Experimental fish were produced from a private pond hatchery in March 2010 and transferred to the Fisheries Science Institute, Chonnam National University, Korea. Black rockfish (mean body weight 10.05 ± 0.44 g, mean body length 8.28 ± 6.94 cm) fed commercial diets (Kurosoi, CP48%, CL12%, Japan) for 3 months were used in the study. The fish were transferred to a 300 L square FRP water tanks, each containing 40 fish, with treatments in triplicate. The diet was given twice a day (08:00, 18:00) ad libitum for 8 weeks. Water that was filtered through a high-pressure sand filter was provided in a running water system at 5 L/min. During the feeding, the fish were kept at a water temperature of 20.2 ± 2.3°C, dissolved oxygen of 6.3 ± 0.4 mg/L, and salt concentration of 32.0 ± 1.2%. The procedure was reviewed and approved by the Chonnam National University Institutional Animal Care and Use Committee (CNU IACUC-2010-45).

Fish bodies were measured before and after the experiments. The experimental fish were starved for 24 h prior to the measurement and the whole body weight of the fish was determined under anesthesia with 100 ppm of the anesthetic for fish only (AQUI-S, New Zealand). In addition, 10 fish randomly picked from each water tank at the beginning and end of each experiment were subjected to body weight, whole length, and body length measurement to calculate the weight gain (WG), feed efficiency (FE), and protein efficiency rate (PER), as growth performance indicators. To investigate the effects of the diet with FSM supplementation on fish body internal organs, the hepatic weight, the viscera weight, and the gut length were determined after dissection, and the viscerasomatic index (VSI), hepatosomatic index (HSI), stomach somatic index (SSI), relative length of the gut (RLG), and condition factor (CF) were calculated via the equations below. The livers and dorsal muscles were isolated for component analyses and then stored at −45°C until further analyses.

- VSI (%) = viscera weight (g) × 100/body weight (g)
- HSI (%) = hepatic weight (g) × 100/body weight (g)
- SSI (%) = stomach weight (g) × 100/body weight (g)
- RLG = gut length (cm)/total length (cm)
- CF = body weight (g) × 100/body length (cm^3^)

Proximate analyses of the experimental diets, livers, dorsal muscles, and whole body were carried out based on AOAC methods (1995). Moisture content was measured using a moisture analyzer with an air-oven method (HR 73 halogen moisture analyzer, Switzerland), and crude protein content was determined by a Kjeldahl nitrogen quantification method (N × 6.25) using an automated analyzer (KJELTEK auto sampler system 1035 analyzer, Switzerland). Crude fat content was analyzed by an ether extraction method via an auto extraction unit (Soxtec 2050 auto extraction unit, Switzerland), and crude ash content was measured using a direct furnace method at 550°C (EYELA Electric furnace TMF-3100, Japan).

### Hematological analysis

Blood composition was analyzed to investigate the health of the experimental fish. After not eating for 24 h at the end of the experiments, blood was collected from the tail blood vessels of 10 fish that were randomly picked in each group using a 1 mL disposable syringe treated with heparin-Na (Sigma, 100,000 units, 2.5 mg/mL), as an anticoagulant. Hemoglobin (Hb, Asan Pharm, Korea) was then measured immediately in whole blood using a commercial kit, and hematocrit (Ht) was measured using a glass capillary. The collected blood was centrifuged for 15 min (12,000 rpm, 4°C), and the separated plasma was subjected to analyses of glucose, total cholesterol, high density lipoprotein (HDL)-cholesterol, glutamic oxaloacetic transaminase (GOT), glutamic pyruvic transaminase (GPT), total protein, and triglyceride using a kit (Asan Pharm, Korea).

Lysozyme activity was measured using a turbidimetric method based on Parry et al. (1965). Briefly, 950 μL of a *Micrococcus lysodeikticus* (0.2 mg/mL) suspension (pH 6.2) was mixed with 50 μL of serum and was reacted at 25°C for 30 s and 4.5 min followed by measuring absorbance at 530 nm. Lysozyme activity was expressed as units/mL, with 1 unit indicating a decrease in absorbance of 0.001/min.

### Stress recovery tests

Based on the study by Ji et al. (2009), the stress recovery rate was measured by an anesthesia test and an air exposure test within 48 h. For the anesthesia test, 10 fish were randomly picked in each experimental group and were given anesthesia in the water bath with 800 ppm of 2-phenoxyethanol (Sigma, USA) for 3 min. The fish were then transferred to running water and the recovery time was measured every 30 s by using a timer, in triplicates for each experimental group. For the air exposure test, 10 fish in each experimental group were randomly collected in a square plastic basket that strains out water and were exposed to the air for 25 min. The fish were then transferred to running water and mortality was measured after 6 h, in triplicates for each experimental group.

To investigate blood composition changes with respect to air exposure stress, 10 fish in each experimental group were randomly collected in a square plastic basket that strains out water and were exposed to the air for 5 min. After being transferred to running water, 2 fish in each experimental group at 1, 2, 4, and 6 h post-treatment were treated with 100 ppm of an anesthetic for fish (AQUI-S, New Zealand) and blood was collected from the tail blood vessels. The blood was centrifuged for 15 min (12,000 rpm, 4°C) and plasma was separated followed by analyzing hematocrit, hemoglobin, glucose, total cholesterol, triglyceride, GOT, and GPT via a commercial kit (Asan Pharm, Korea).

### Statistical analysis

Statistical analyses of the results were performed using an ANOVA-test. The significance of the means was tested by a Duncan’s multiple range test (Duncan 1955) in the SPSS statistical program.

## Results

### Growth performance

The growth performance of the black rockfish fed diets with different levels of flounder skin meal (FSM) for 8 weeks is shown in Table 2. The survival rate was 100% in all groups. WG and SGR were significantly greater for FSM 20% than the control (*P < 0.05*). The feed intake (FI) was significantly greater for FSM 20% than the control (*P < 0.05*). For FE, no significant differences were found between the control group and the FSM supplement groups, and FSM 20% was observed to be significantly higher than FSM 10% (*P* < *0.05*). The protein intake (PI) was significantly greater for FSM 20% than the control (*P < 0.05*). The protein efficiency rate (PER) was not significantly different among any of the experimental groups.

**Table 2:** **Growth performance of black rockfish,** ***S. schlegeli*** **fed the test diets with various levels of FSM for 8 weeks**

	FSM level (%)			
	Control (0)	5	10	20
IBW (g)^1^	10.90 ± 0.37	10.60 ± 0.12	10.47 ± 0.17	10.92 ± 0.45
FBW (g)^2^	33.12 ± 1.54^ab^	32.22 ± 1.72^ab^	30.56 ± 0.64^a^	33.72 ± 1.98^b^
SR (%)^3^	100^ns^	100	100	100
WG (%)^4^	185.35 ± 6.06^a^	203.87 ± 14.37^ab^	191.98 ± 10.51^ab^	208.71 ± 6.97^b^
SGR (%)^5^	0.36 ± 0.02^a^	0.39 ± 0.03^ab^	0.36 ± 0.01^a^	0.41 ± 0.03^b^
FI (g)^6^	31.12 ± 0.45^a^	31.76 ± 1.02^a^	31.17 ± 0.45^a^	36.58 ± 1.03^b^
FE (%)^7^	60.23 ± 3.56^ab^	64.40 ± 4.88^ab^	59.85 ± 2.35^a^	67.94 ± 4.65^b^
PI (g)^8^	17.43 ± 0.25^a^	17.78 ± 0.57^a^	17.45 ± 0.25^a^	20.31 ± 0.59^b^
PER (%)^9^	1.16 ± 0.06^ns^	1.21 ± 0.06	1.15 ± 0.04	1.12 ± 0.05

Data are mean ± standard deviation of three group of fish (n = 45). Values with different superscripts are significantly different (*P* < 0.05).

ns not significant.

^1^ Initial mean body weight.

^2^ Final mean body weight.

^3^ Survival rate.

^4^ Weight gain: (final body weight - initial body weight/initial body weight) × 100.

^5^ Specific Growth rate (%): (final body weight - initial body weight)/the time interval in days × 100.

^6^ Feed intake.

^7^ Feed efficiency (%): (fish weight gain/feed intake) × 100.

^8^ Protein intake.

^9^ Protein efficiency rate (%): (fish weight gain/protein intake) × 100.

The CF, VSI, HSI, SSI, and RLG of the black rockfish fed diets with different levels of FSM for 8 weeks are shown in Table 3. Significant differences were not observed in CF, VSI, HIS, SSI, and RLG between the control group and the FSM supplement groups.

**Table 3:** **Condition factor (CF), viscerasomatic index (VSI), hepatosomatic index (HSI), stomach somatic index (SSI), and relative length of gut (RLG) of black rockfish,** ***S. schlegeli*** **fed the test diets with various levels of FSM for 8 weeks**

	FSM level (%)			
	Control (0)	5	10	20
CF	3.11 ± 0.08^ns^	2.95 ± 0.27	2.88 ± 0.20	2.91 ± 0.33
VSI	10.76 ± 1.27^ns^	11.29 ± 0.90	11.48 ± 3.03	11.01 ± 1.20
HSI	3.35 ± 0.61^ns^	3.61 ± 0.35	3.56 ± 0.76	3.07 ± 0.22
SSI	1.00 ± 0.19^ns^	1.12 ± 0.17	1.07 ± 0.07	1.22 ± 0.53
RLG	0.89 ± 0.14^ns^	0.93 ± 0.08	0.94 ± 0.13	0.97 ± 0.09

Data are mean ± standard deviation of three group of fish (n = 10). Values with different superscripts are significantly different (*P* < 0.05).

ns not significant.

### Proximate analysis

Proximate analysis results of livers, dorsal muscles, and whole fish bodies are shown in Table 4.

**Table 4:** **Proximate analysis of whole body, liver, and dorsal muscle of black rockfish,** ***S. schlegeli*** **fed the test diets with various levels of FSM for 8 weeks (wet matter)**

		FSM level (%)			
		Control (0)	5	10	20
Moisture (%)	whole body	69.07 ± 0.35^ab^	68.82 ± 0.58^ab^	68.26 ± 0.68^a^	69.51 ± 0.63^b^
	liver	47.55 ± 1.37^a^	46.00 ± 0.13^a^	46.26 ± 0.25^a^	51.72 ± 1.18^b^
	dorsal muscle	72.79 ± 2.17^ns^	70.47 ± 0.78	73.03 ± 1.44	71.74 ± 1.08
Crude protein (%)	whole body	14.47 ± 0.42^b^	14.73 ± 0.05^bc^	15.08 ± 0.01^c^	14.06 ± 0.05^a^
	liver	8.37 ± 0.06^a^	8.74 ± 0.12^b^	8.77 ± 0.05^b^	8.67 ± 0.01^b^
	dorsal muscle	18.13 ± 0.07^a^	19.70 ± 0.06^d^	18.82 ± 0.05^c^	18.39 ± 0.02^b^
Crude lipid (%)	whole body	9.37 ± 0.62^ns^	9.47 ± 1.46	9.53 ± 0.24	7.76 ± 1.80
	liver	29.59 ± 1.04^b^	26.72 ± 0.96^ab^	26.53 ± 2.68^ab^	23.39 ± 2.59^a^
	dorsal muscle	6.17 ± 0.79^ns^	5.12 ± 1.93	4.68 ± 0.16	6.53 ± 0.82
Ash (%)	whole body	3.81 ± 0.08^ns^	3.85 ± 0.03	3.92 ± 0.06	3.83 ± 0.07
	liver	0.65 ± 0.07^a^	0.76 ± 0.07^a^	0.95 ± 0.06^b^	1.10 ± 0.07^c^
	dorsal muscle	1.30 ± 0.05^ns^	1.37 ± 0.08	1.34 ± 0.02	1.29 ± 0.24

Data are mean ± standard deviation of three group of fish (n = 3). Values with different superscripts are significantly different (*P < 0.05*).

ns not significant.

Whole body moisture showed no significant differences between the control group and the FSM supplement groups, while FSM 20% was significantly higher than the FSM 10% group (*P < 0.05*). Liver moisture in FSM 20% was significantly higher than in the control, FSM 5%, and FSM 10% groups (*P < 0.05*). In the dorsal muscles, there were no significant differences between the control group and the FSM supplement groups.

Whole body crude protein was significantly higher in the 10% FSM group than the control, FSM 20% groups (*P < 0.05*). In the livers, the FSM groups were significantly higher than the control group (*P < 0.05*). The dorsal muscles were found to be significantly higher in the FSM groups than the control group. Among FSM groups, FSM 5% was significantly higher than FSM 10% and FSM 20% (*P < 0.05*).

In the crude lipid contents of whole body and dorsal muscle, there were no significant differences between the control and FSM supplement groups. Liver lipid was significantly higher in the control group than the FSM 20% (*P < 0.05*).

No significant differences in the whole bodies and dorsal muscles were found between the control and FSM supplement groups. Liver ash was significantly higher in the FSM 10% and FSM 20% groups than the control and FSM 5% groups (*P < 0.05*).

### Hematological analysis

Hematocrit (Ht), Hemoglobin (Hb), glucose, GOT, GPT, triglyceride, total cholesterol, HDL-cholesterol, and total protein levels are shown in Table 5. The Ht, Hb, glucose, triglyceride, GOT, GPT, total cholesterol, and HDL-cholesterol levels did not show significant differences between the experimental groups. Total protein was significantly higher in the FSM 20% group than the control, FSM 5%, and FSM 10% groups (*P < 0.05*).

**Table 5:** **Hematocrit (Ht), Hemoglobin (Hb), glucose, triglyceride, GOT, GPT, total cholesterol, HDL-cholesterol, and total protein of black rock fish,** ***S. schlegeli*** **fed the test diets with various levels of FSM for 8 weeks**

	FSM level (%)			
	Control (0)	5	10	20
Ht (%)	36.00 ± 0.89^ns^	35.83 ± 1.17	34.33 ± 1.97	34.50 ± 2.26
Hb (g/dL)	8.82 ± 1.26^ns^	8.79 ± 0.62	8.73 ± 0.81	8.54 ± 0.85
Glucose (mg/dL)	4.76 ± 2.37^ns^	4.56 ± 2.76	3.42 ± 1.24	3.65 ± 2.20
Triglyceride (mg/dL)	408.96 ± 76.02^ns^	643.88 ± 188.10	481.79 ± 189.42	498.21 ± 16.64
GOT (Karmen/mL)	42.54 ± 26.82^ns^	71.53 ± 18.61	39.00 ± 18.03	50.43 ± 4.80
GPT (Karmen/mL)	44.29 ± 8.21^ns^	49.23 ± 7.63	43.03 ± 13.23	45.78 ± 10.63
Total cholesterol (mg/dL)	213.89 ± 67.66^ns^	289.84 ± 40.48	251.71 ± 50.72	294.98 ± 65.01
HDL-cholesterol (mg/dL)	202.30 ± 26.07^ns^	210.03 ± 32.18	213.99 ± 34.30	209.84 ± 4.55
Total protein (g/dL)	3.27 ± 0.13^a^	2.90 ± 0.34^a^	3.33 ± 0.52^a^	4.34 ± 0.44^b^

Data are mean ± standard deviation of three group of fish (n = 10). Values with different superscripts are significantly different (*P* < 0.05).

ns not significant.

**Figure 1:**
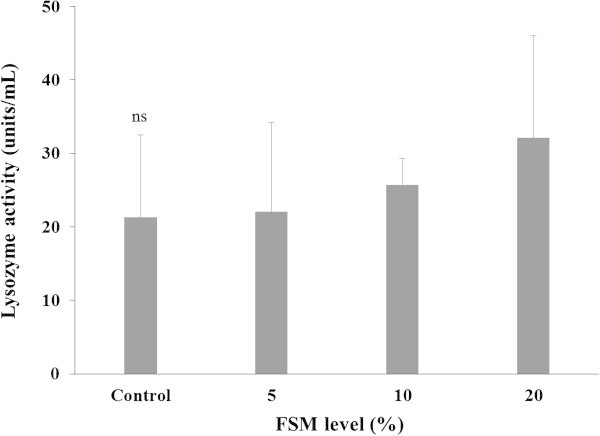
**Lysozyme activity of black rockfish,** ***S. schlegeli*** **fed the test diets with various levels of FSM for 8 weeks.** Bar indicates standard deviation (n = 3). ns not significant.

Lysozyme activity in the plasma is shown in Figure 1. The lysozyme activity tended to be higher in FSM supplement groups than the control group, but the differences were not significant.

### Stress recovery tests

**Figure 2:**
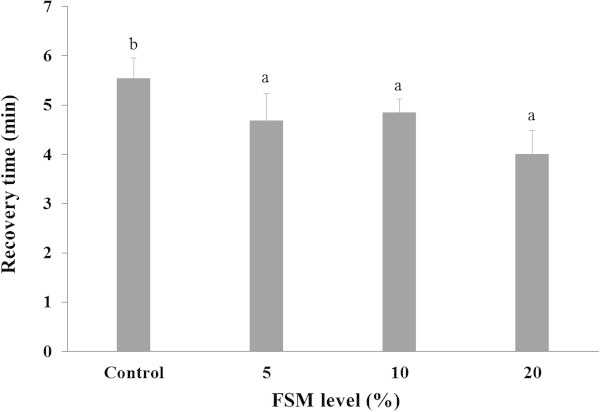
**Recovery time of black rockfish,** ***S. schlegeli*** **fed the test diets with various levels of FSM for 8 weeks after the anesthesia test.** Bar indicates standard deviation (n = 3). Bars with different letters differ significantly (P < 0.05).

Recovery time after anesthesia with 2-phenoxyethanol is shown in Figure 2. The recovery time after the anesthesia was in a range of 3.5–6.0 min. All FSM supplement groups were significantly faster than the control group (*P < 0.05*), and FSM 20% group had the fastest recovery time.

**Figure 3:**
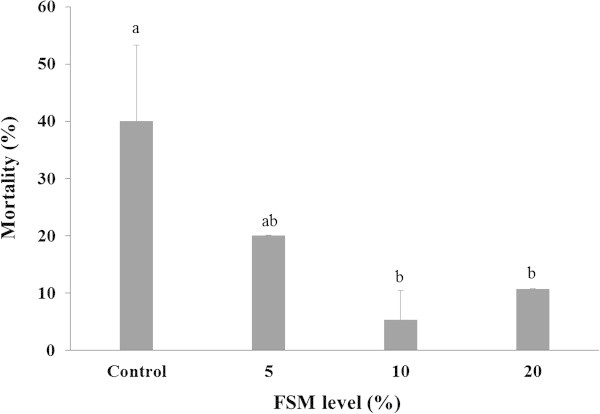
**Mortality of air exposure test on the black rockfish,** ***S. schlegeli*** **fed the test diets with various levels of FSM for 8 weeks.** Bar indicates standard deviation (n = 3). Bars with different letters differ significantly (P < 0.05).

Mortality after exposure to the air is shown in Figure 3. The mortality was not significantly different in FSM 5% and the control group, whereas it was significantly lower in FSM 10% and FSM 20% compared to the control (*P < 0.05*).

**Figure 4:**
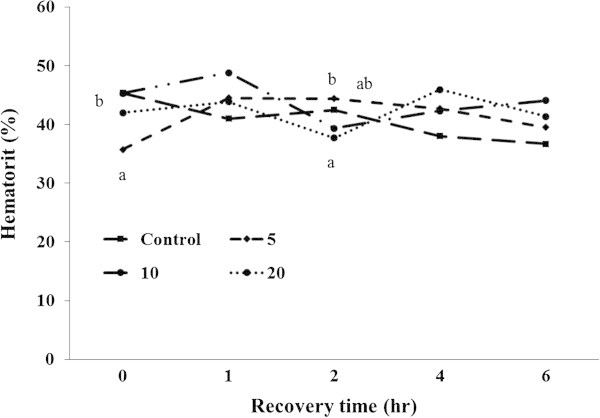
**Changes in plasma hematocrit level of 5-min air exposure test on black rockfish,** ***S. schlegeli*** **fed the test diets with various levels of FSM for 8 weeks.** Different letters differ significantly (P < 0.05).

**Figure 5:**
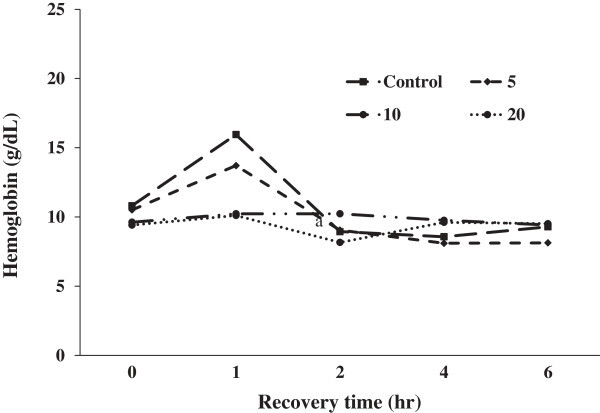
**Changes in plasma hemoglobin level of 5-min air exposure test on black rockfish,** ***S. schlegeli*** **fed the test diets with various levels of FSM for 8 weeks.** Different letters differ significantly (P < 0.05).

**Figure 6:**
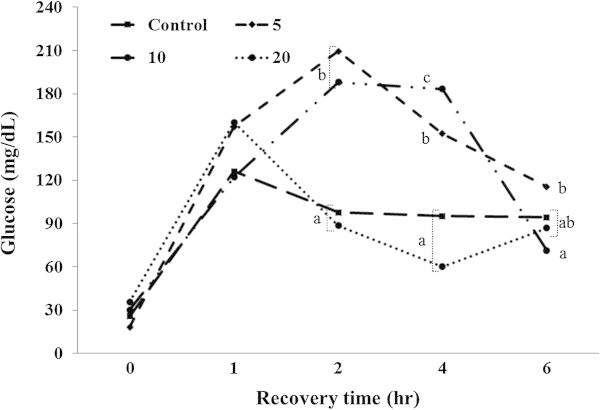
**Changes in plasma glucose level of 5-min air exposure test on black rockfish,** ***S. schlegeli*** **fed the test diets with various levels of FSM for 8 weeks.** Different letters differ significantly (P < 0.05).

**Figure 7:**
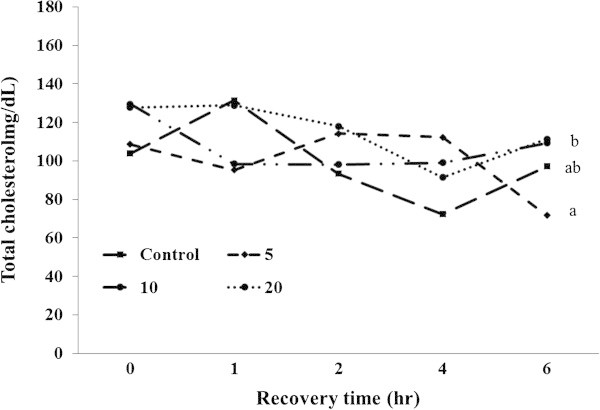
**Changes in plasma total cholesterol level of 5-min air exposure test on black rockfish,** ***S. schlegeli*** **fed the test diets with various levels of FSM for 8 weeks.** Different letters differ significantly (P < 0.05).

**Figure 8:**
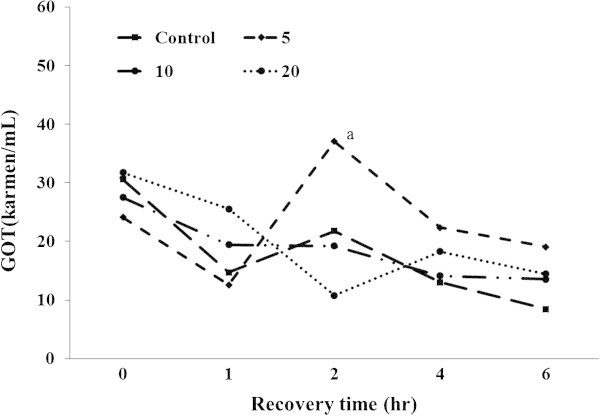
**Changes in plasma GOT level of 5-min air exposure test on black rockfish,** ***S. schlegeli*** **fed the test diets with various levels of FSM for 8 weeks.** Different letters differ significantly (P < 0.05).

**Figure 9:**
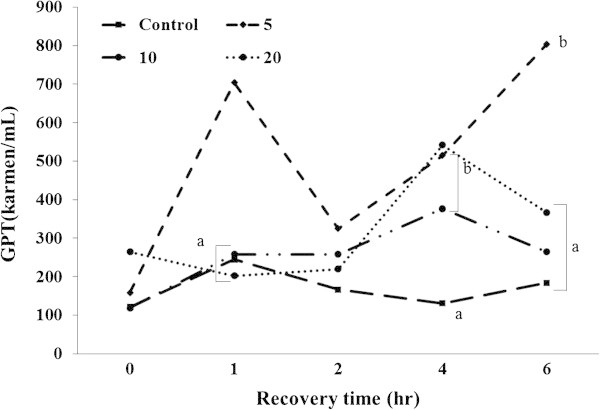
**Changes in plasma GPT level of 5-min air exposure test on black rockfish,** ***S. schlegeli*** **fed the test diets with various levels of FSM for 8 weeks.** Different letters differ significantly (P < 0.05).

Ht, Hb, glucose, total cholesterol, GOT, and GPT were analyzed during recovery time (Figures 4, 5, 6, 7, 8, 9).

At 1 h after the air exposure, the Ht level of FSM 10% was the highest and followed to FSM 5% and FSM 20%, and the control group was the lowest, but the difference was not significant. At 2 h after the air exposure, there were no significant differences observed between the control group and the FSM supplement groups while FSM 5% was significantly higher than FSM 20% (*P < 0.05*). The FSM groups tended to be higher than the control group at 4 h after the exposure, and all Ht of the experimental groups maintained about 40% at 6 h after the exposure, but there were no significant differences either 4 or 6 h after the exposure (Figure 4).

The Hb was not significantly different among the groups at 1 h after the air exposure although it rapidly increased in the control and FSM 5% groups. The control group showed the greatest increase of Hb. At 2 h after the exposure, significant differences were not found among the groups, and the Hb level had remarkably decreased to what it was before the exposure in all groups except for FSM 10% and FSM 20%. At 4 h after the exposure, FSM 20% showed an increase in the Hb level to where it was before the exposure. Significant differences were not observed at 6 h after the exposure and the Hb levels were maintained at the same levels as before the exposure (Figure 5).

At 1 h after the exposure, the glucose levels showed no significant differences among the groups and had remarkably increased in all groups. At 2 h after the exposure, glucose level was significantly higher in the FSM 5% and FSM 10% groups than the control and FSM 20% groups (*P < 0.05*). At 4 h after the exposure, the glucose levels in FSM 10% and FSM 5% were observed to be significantly higher than the control and FSM 20% groups (*P < 0.05*). At 6 h after the exposure, significant differences were present between the groups. However, the glucose level had notably decreased in the FSM 10% and FSM 5% groups and was maintained in control and FSM 20% groups (Figure 6).

The total cholesterol at 1 h after the exposure was not significantly different among the groups, but had increased in control and decreased in the FSM 5% and FSM 20%. Significant differences were also not observed at 2 h after the exposure, and the total cholesterol tended to have decreased in all groups except for FSM 5%. At 4 h after the exposure, there were no significant differences among the groups, although it had decreased in the control group and FSM 20%, and increased in FSM 10%. At 6 h after the exposure, significant differences were not found between control and FSM supplement groups, while significantly higher total cholesterol was observed in FSM 10% and FSM 20% than the FSM 5% (*P < 0.05*) (Figure 7).

GOT showed no significant differences among the groups at 1 h after the exposure although it had decreased in all groups. At 2 h after the exposure, FSM 5% had markedly increased, and significantly higher than control, FSM 10%, and FSM 20% groups. At 4 h after the exposure, GOT had decreased in all groups except for FSM 20% and was higher in FSM 5% and FSM 20%. Decreased GOT was observed in all groups at 6 h after the exposure, and significant differences were not observed in all groups (Figure 8).

GTP was significantly higher in FSM 5% than the control, FSM 10%, and FSM 20% groups at 1 h after the exposure. At 2 h after the exposure, decreased GTP was found in FSM 5%, and there were no significant differences among the groups. Noticeably increased GPT was observed in all groups except for control at 4 h after the exposure, and FSM supplement group were significantly higher than the control group. GPT increased in FSM 5% but decreased in the other groups at 6 h after the exposure. Especially, GTP was significantly higher in FSM 5% compared to the control group, FSM 10%, and FSM 20% (*P < 0.05*) (Figure 9).

## Discussion

Due to increased prices and unstable supplies of fishmeal, there has been extensive research on alternate protein sources for fish feed (Barrows and Hardy 2000). Not only have the biochemical properties of these alternative protein sources been studied, but also their feasibility through direct feeding studies in fish (Kim and Bai 1997; Lee and Yoo 1996; Lee et al. 2010; Pham et al. 2005; Shapawi et al. 2007; Toppe et al. 2006; Uyan et al. 2006).

In our previous study, biochemical analyses of fish skin from four species of fish (*P. olivaceus*, *S. schlegeli*, *L. maculates*, and *P. major*) were performed (Cho et al. 2014). The crude protein content of the skin of these fish ranged from 73% to 94% by dry weight; this high level was partly due to a high content of structural protein, collagen. Among the four species, *P. olivaceus* had the thickest dermal and epidermal layers in the dorsal skin. This species was also associated with the highest extraction ratio of acid-soluble collagen. The ASC ratio of the olive flounder skin (20.69%) was highest, followed by that of the red sea bream (20.44%), sea bass (14.74%), and black rockfish (11.00%) based on dry weights. We also examined whether fish skin could be a cost-effective alternative to current fish meal sources. Our analysis indicates that, when it is supplemented with additional fish oils and essential amino acids, fish skin is a viable alternative for fish meal formulations.

According to Kim and Bai (1997), when a fishmeal mixed with animal protein sources including blood meal, squid liver meal, meat and bone meal, greaves, feather meal, and essential amino acids that were gradually substituted for to 20–60% of the regular diet was fed to black rockfish for 6 weeks, growth performance did not significantly change at concentrations up to 40% of the fishmeal. In addition, once black rockfish were fed the fishmeal mixed with 12.5–50% animal protein supplement for 16 weeks, up to 12.5% of the fishmeal could be substituted before the rate of gain and feed efficiency started showing large differences as the level of supplement increased. This was reported to be because of diet intake preferences, *in vivo* enzyme activity, and metabolism (Kim and Bai 1999). In the present study, although significant differences were not found between the control group and the FSM supplement groups, it tended to decrease gradually with an increase of the supplement. Such an FSM supplement at the same fishmeal ratio seems to be capable of facilitating the preference and metabolism of fish, and vitamin C addition would provide synergetic effects in the FSM supplement. Aksnes et al. (2008) added 0.7–5.6 g/kg of hydroxyproline to vegetable protein diets and fed them to salmon (*Salmo salar* L.) for 88 days. They found that adding 2.9 g/kg of hydroxyproline to the diet increased the growth of the salmon by approximately 14%. The diet from the current study is also expected to improve the growth performance because the FSM used in this study contains abundant hydroxyproline, which is a collagen-specific amino acid.

Meanwhile, the condition factor (CF), viscerasomatic index (VSI), and hepatosomatic index (HSI) were significantly lower in the FSM supplement groups. In particular, CF was observed to be significantly lower in the FSM supplement groups compared to the control group. When black rockfish were fed a diet with fishmeal supplement mixed with animal proteins, Kim and Bai (1997,1999) observed that HSI increased in 2.8 g juvenile fish with an increase in the supplement, and HIS and CF decreased in 21.1 g juvenile fish, which indicates discrepant size-dependent results, even in the same kind of fish. However, when using soybean meal as an alternative fishmeal, decreased HIS and CF were found in juvenile *Paralichthys olivaceus* in a dose-dependent manner (Kim et al. 2000b). HIS was not significantly different in *Hexagrammos otakii* Jordan et Starks when soybean meal and feather meal were added gradually as fishmeal replacement (Lee and Lee 1998). Meanwhile, hybrid striped bass (*Morone chrysops* ♀ × *M. saxatilis* ♂) fed diets with some added poultry by-products as protein sources to replace fishmeal showed significantly increased HIS and intraperitoneal fat (IPF) when up to 70% of the fishmeal was substituted (Rawles et al. 2006). Additionally, high fat content in the fish body was found in silver seabream (*Rhabdosargus sarba*) and Nile tilapia (*Oreochromis niloticus* L.) with the addition of poultry by-products (El-Sayed 1994,1998). It was also reported that body fat and HIS (Steffens 1994) and VSI and HSI (Zoccarato et al. 1996) increased in *Oncorhynchus mykiss* in response to poultry by-products supplementation. Comparing the results of such fishmeal substitution is difficult because of the various protein sources and fish utilized. However, the nutritional characteristics of the alternative protein sources affect lipid metabolism in the body such that HSI, CF, and VSI would be influenced. In particular, as FSM contains relatively higher lipids and proteins compared to fishmeal, it would affect lipid accumulation and metabolism. In the FSM supplement group, the moisture and protein content in the whole body increased and the lipid content decreased, and the ash content decreased significantly compared to the control group. Such results were in agreement with the moisture and protein content in muscles, and the liver showed the same results with the protein and ash content in the whole body. However, the lipids in the liver were confirmed to be higher than the control group, indicating that lipid accumulation in the liver increased. The body composition of fish is affected by various factors such as intraspecific strain differences, water temperature, and increased body weight, and is influenced the most by the amount of feed supplied and the mix proportions of the feed (Nandeesha et al. 1995; Zeitler et al. 1984). It was also reported that lipids and moisture in fish bodies decreased with fish growth while protein and mineral content mostly remained unchanged (Murai et al. 1985). Kim and Bai (1997,1999) reported that feeding black rockfish with an animal protein supplement had no effects on body composition unless there were nutritional problems, which differs from this study. However, Bai et al. (1998) found that when *Cyprinus carpio* were fed diets with added greaves and meat and bone meal containing over 2 times more crude fat than fishmeal, the crude fat accumulated in the body was used as an energy source, thereby increasing protein accumulation. This is in agreement with the present study that added FSM (17%) possessing over 2 times higher lipid content compared to white and brown fishmeal (7–8%). Even though black rockfish and *Cyprinus carpio* live in different habitats and the composition of the greaves, meat and bone meal, and FSM are different, the lipid content in the FSM seems to be used as energy sources because both fishes use lipids as an energy source. This was partially confirmed by the increased crude protein content and the decreased lipid content in the whole fish and muscles. Rogie and Skinner (1985) found that lipoproteins of rainbow trout were synthesized in the liver cells, and Lee et al. (2000) mentioned that inhibited lipoprotein synthesis owing to the impaired metabolism of liver cells could result in lipid accumulation in the liver without using them in the liver in black rockfish. In the current study, HIS was decreased with respect to the FSM supplementation, but the lipids in the liver were increased and tended to accumulate. It is difficult to conclude whether such results affect lipoprotein metabolism due to the FSM supplement in the feed. However, based upon the high lipid content in the liver of the control group, there seems to be relationships between FSM supplementation and vitamin C addition. Further studies need to be done on this topic.

This study observed no significant differences in hematocrit, hemoglobin, glucose, GOT, GPT, total cholesterol, or HDL-cholesterol in the blood components. The lysozyme activity was not significantly different among the FSM supplement groups and the control group. However, the triglycerides tended to decrease, and the total protein in FSM 20% was found to be significantly higher. Changes in the plasma constituents are critical indicators of the health status of organisms since they can differ due to a lack of essential nutrients in feed (Murai et al. 1982a) or the feeding environment of the fish (Park et al. 1999). In general, hemoglobin levels of healthy fish are known to be 9–10 g/dl (Post 1983). Hemoglobin and hematocrit levels of 21.4 g black rockfish fed a fishmeal replacement were reported to be 7.3–9.0 g/dl and 40.2–45.4%, respectively. These were slightly lower and higher, respectively, than the hemoglobin and hematocrit levels in the present study, indicating that the adequate amount for fish is still not clear (Kim and Bai 1999).

Lysozymes, lymphocyte-derived mucous bacteriolytic enzymes with antibiotic-like characteristics, are widely distributed in nature and have been found in secretions such as mucous, saliva, and blood. Lysozymes dissolve acetyl-monopolysaccharides in the cell walls of gram-positive bacteria and show direct bacteriolytic action in gram-negative bacteria. In accordance with the complement and antibody supports, the bacteriolytic actions are also improved, which are complement-dependent. Therefore, lysozymes are closely related to the complement and become a part of the intrinsic defense mechanism against parasitic, bacterial, and viral infections in a number of animals (Ingram 1980; Lim et al. 2009). In this study, lysozymes did not show significant differences among the FSM supplement groups and the control group. However, lysozymes tended to increase, so it is possible that they are affected by the FSM. Vitamin C increases resistance to diseases in fish (Verlhac and Gabauda 1994,1997). Therefore, synergetic effects with the FSM supplement are also expected in the presence of long-term feeding.

There are various factors that induce stress in fish. In particular, the handling, sorting, shipment, and transportation necessary during fish farming and bad water quality stress fish (Barton and Iwama 1991; Schreck 1982; Wendelaar Bonga 1997). In fish farming and the fishery industry, various harvesting methods (Harman et al. 1980; Hopkins and Cech 1992; Maule and Mesa 1994; Maule et al. 1989; Mitton and McDonald 1994), physiological shock (Gadomski et al. 1994; Sverdrup et al. 1994), and exposure to contaminated environments (Marshall Adams 1990; Brown 1993; Cairns et al. 1984; Folmar 1993; Kime 1995; Niimi 1990) induce stress (Iwama et al. 1997). Stress affects every part of the fish from community structures to biochemical instability ((Marshall Adams 1990) and is also a cause of mass mortality in fish farming (Iwama et al. 1997). In the present study, the FSM supplement groups showed fast recovery in the anesthesia and air exposure tests. Ji et al. (2007) observed fast recovery after anesthesia and positive effects regarding the air exposure in juvenile *Paralichthys olivaceus* fed diets supplemented with medicinal herbs for 8 weeks. This was suggested to be because of improvements in liver functions and active biosynthesis of glycogen owing to the addition of herbs with antioxidant properties. Similar results were also confirmed in juvenile *Pagrus major* fed medicinal herbs for 12 weeks. This was also ascribed to the medicinal herbs containing antioxidants that prevent oxidative damages inducing aging, functional impairments, and diseases (Ji et al. 2009). The skin of *Paralichthys olivaceus* used in this study is a protein source containing lots of collagen, but no antioxidant functions of collagen have been reported. To study the prevention of collagen destruction, collagen from squid skin was added to human dermal cells, cultured for 24 h, and analyzed through UV for 24 h (Kwon et al. 2008). This treatment also accelerated differentiation by facilitating the growth of cell lines cultured with type I collagen (Kim et al. 2000a). In the human body, melanin, which raises pigmentation such as spots, freckles, and age spots in the presence of excess production, thereby promoting skin damage, is produced during the biosynthesis of tyrosinase (Invergar and McEvily 1992), and a study confirmed tyrosinase inhibition in the type 1 collagen of calves and the collagen extracted from squid skin (Kwon et al. 2008). This tyrosinase inhibitory activity is associated with phenol content and antioxidant effects (Ra et al. 1997), and the small phenols in collagen were reported to be the major cause of tyrosinase inhibition (Kwon et al. 2008) in a study that did not mention antioxidant effects. Matsuda et al. (2006) reported that skin fibroblast density and collagen density significantly increased in young pigs fed collagen peptides. Because the previous studies were mostly performed at the cell level or with terrestrial animals, it is difficult to make direct comparisons. However, the factors related to collagen are expected to have positive effects on cell aging, especially the antioxidant properties. According to Walrand et al. (2008), when dairy products with added collagen hydrolysates were given to 15 men, the concentration of blood amino acids related to collagen greatly increased and collagen synthesis was facilitated. Even through the results were obtained from humans, collagen is considered to influence blood biochemical factors and energy metabolism as this study also found a significant decrease in blood lipids and an increase in blood total proteins. However, since there are no studies on collagen intake in aquatic organisms like fish, systematic studies regarding the intake of fish and factors related to collagen are necessary in near future.

Schreck (1982) mentioned that blood lactic acid, lipids, electrolytes, hemoglobin (Hb), total protein, hematocrit (Ht), and liver glycogen could be used to determine the presence or absence of stress. In addition to the stress indicators, such items are indicators used in the evaluation of health in fish bodies (Wedemeyer and Mcleay 1981; Wedemeyer and Yasutake 1977). Following the air exposure test in this study, stress responses included a rapid increase in hemoglobin (Hb) 1 h after exposure. Significant differences were not observed among the experimental groups, although the increase in the FSM groups was smaller than that in the control group. Generally, Ht and Hb are known to be indicators of oxygen transportation in the body (Min et al. 2003). Stress in marine fish was reported to increase blood Ht and Hb levels (Davis and Parker 1990). In this study, however, Ht did not change considerably, and Hb tended to increase. Such a result is considered to be due to the differences in the response time of Hb rather than Ht. Further, as the air exposure test was directly related to oxygen consumption, oxygen was rapidly consumed within 2 h. The recovery rate seems to have differed due to the rapid supplementation of hemoglobin, especially in the FSM groups compared to the control group.

Generally, blood glucose concentrations are elevated by stress (Olsen et al. 1995). Barton and Iwama (1991) mentioned that increased glucose levels with an increase in cortisol levels were the result of a second reaction owing to stress hormones. Ji et al. (2009) reported that the rapid recovery of blood cortisol and glucose to the levels from before the air exposure test was observed in the medicinal herb supplement group, which showed significant growth when juvenile *Pagrus major* fed with medicinal herb supplemented diets were exposed to the air. This was suggested to be because of the rapid reduction of consumption of the stored energy owning to stress. In the present study, we also observed an increase in the glucose concentration in all of the experimental groups due to drastic energy consumption owing to stress, and rapid recovery tended to be exhibited in the groups with high weight gain, which was in agreement with the results of Ji et al. (2009). The stress is capable of inhibiting growth by consuming energy stored in the body due to liver gluconeogenesis and increased aminotransferase activity as well as protein catabolism (Davis et al. 1985).

The total cholesterol also tended to be decreased in all experimental groups after the air exposure, which indicates that energy consumption due to increased metabolism occurs in lipids.

The amine transferases GOT and GPT are distributed in blood in the liver and spleen of fish bodies. While they maintain low activity when the fish body is healthy, they are released in the presence of tissue necrosis or diseases, resulting in increased activity (Min et al. 2003). GOT was observed to increase in trout when stress was induced with rapid changes of the salinity in the feeding water (Chang and Hur 1999). Juvenile black sea breams also showed increased GOT with acute salt stress (Min et al. 2003). Significantly increased GOT was found in adult and juvenile *Paralichthys olivaceus* in response to acute stress from low salt levels in the feeding water (Her et al. 2002). Her et al. (2002) reported that this was due to impaired liver functions owing to physiological burdens on liver and spleen cells and excess energy consumption. However, although we observed a decrease in GOT and an increase in GPT, results that differed from previous studies, we were able to confirm that the stress due to air exposure negatively influenced liver functions. The FSM groups had especially high GPT levels compared to the control group. Even though such a difference is considered to mainly be caused by vitamin C concentrations, it showed higher values in the supplement group with greater than FSM10% than the control group. It is therefore expected that FSM functional substances such as collagen have positive effects that promote the quick recover of energy metabolism following impaired liver functions.

In this study, the black rockfish was fed a diet in which 5%, 10%, or 20% of the casein was substituted with FSM with vitamin C levels of 400 mg/kg for 8 weeks. Significantly higher growth performance and feeding efficiency were observed in FSM 20% compared to the casein supplement group. CF, HSI, and VSI levels and lipid content in the body were lower while protein accumulation increased. Essential amino acids in the body also tended to increase, and amino acids and free amino acids showed different patterns to each other and confirmed that FSM supplementation influenced amino acid composition. There were also differences observed in organic acids and free sugars. Blood composition, lysozyme activity, and stress responses were investigated to confirm the health status of the fish, and it was found that stress decreased blood lipids and increased blood proteins. Significant differences were not found in the lysozyme activity, but it tended to increase with FSM supplementation. In the air exposure and anesthesia tests, the stress response was significantly higher in the FSM supplement group, and the FSM groups also showed rapid recovery after air exposure. Taken together, FSM are superior protein sources for the black rockfish compared to purified proteins like casein, and health improvements in the farmed fish are also expected. However, the discrepant results of the vitamin C supplementation indicate that detailed studies are required regarding the relationship between FSM and vitamin C.
